# The Effect of Interventional Program on Breastfeeding Self-Efficacy and Duration of Exclusive Breastfeeding in Pregnant Women in Ahvaz, Iran

**DOI:** 10.1155/2014/510793

**Published:** 2014-08-19

**Authors:** Somayeh Ansari, Parvin Abedi, Shirin Hasanpoor, Soheila Bani

**Affiliations:** ^1^Midwifery Department, Reproductive Health Promotion Research Center, School of Nursing and Midwifery, Jundishapur University of Medical Sciences, Ahvaz 6135715794, Iran; ^2^Community Nutrition, Midwifery Department, Reproductive Health Promotion Research Center, School of Nursing and Midwifery, Jundishapur University of Medical Sciences, Ahvaz 6135715794, Iran; ^3^Midwifery Department, School of Nursing and Midwifery, Tabriz University of Medical Sciences, Tabriz 5157943598, Iran

## Abstract

*Objective*. This study aimed to determine the effect of educational program on Breastfeeding self-efficacy and duration of exclusive breastfeeding in pregnant women in Ahvaz, Iran. *Methods*. This randomized controlled trial was conducted on 120 nulliparous pregnant women who tended to breastfeed. The primary self-efficacy scores of samples were measured using Faux and Dennis breastfeeding self-efficacy questionnaire. Women were randomly recruited into two intervention and control groups. Educational program (two training sessions, each lasted two hours) with two days interval was performed for intervention group. One month after delivery, self-efficacy scores were determined. Six months after child birth, duration of exclusive breastfeeding was assessed. Data were analyzed by means of descriptive and inferential statistics. *Findings*. The breastfeeding self-efficacy in the intervention group increased significantly compared to the control group one month after delivery (123.6 versus 101.7, *P* < 0.001). The duration of exclusive breastfeeding was significantly higher in the intervention group (5.03 mo versus 2.7 mo, *P* < 0.001). Also, there was a significant relationship between breastfeeding self-efficacy and duration of exclusive breastfeeding (*P* < 0.001). *Conclusion*. The educational program could increase the self-efficacy and exclusive breastfeeding duration of mothers. These results can draw the attention of authorities to the importance of educational programs for mothers regarding the exclusive breastfeeding.

## 1. Introduction

Breastfeeding is one of the most important ways to improve the children health and a basic strategy in providing the child growth and survival. For years, the positive effects of breastfeeding on child development and growth as well as maternal health have been well documented [1, 2]. The global recommendation of World Health Organization (WHO) before 2001 was exclusive breastfeeding between 4 and 6 months after birth [3]. In 2002 a systematic review on exclusive breastfeeding showed that infants who breastfed for 4 months had more morbidity and mortality than those with 6 months [4]. Therefore the WHO recommended exclusive breastfeeding until 6 months after birth [5]. Many studies have been done on exclusive breastfeeding that show that the exclusive breastfeeding is still far away from the recommendations of the WHO [5]. A study on 63,071 infants with age less than 24 months in 30 provinces in Iran showed that exclusive breastfeeding at 4 and 6 months after birth was 56.8% and 27.7%, respectively [6]. Another study in Omidieh, Iran, on 400 infants with age 6–18 months showed that the prevalence of exclusive breastfeeding was 61.6% [7]. A study in England showed that infants' exclusive breastfeeding was very low, and only 25% of babies remain breastfed until 6 to 8 weeks after birth and 16% of mothers continued breastfeeding for 3 to 5 months after birth [8]. Also, a recent study in Japan showed that although 96% of mothers were willing to breastfeed their infants, only 44% had exclusive breastfeeding for the first 4 weeks after childbirth. Less than 35% of Canadian mothers and 29% of American mothers had exclusive breastfeeding up to four months. In Canada, one third of women breastfeed their infants and wean their children eight weeks after birth [9]. Considering that the benefits of breastfeeding for infants will increase with the increase of duration of breastfeeding and exclusive breastfeeding, the factors of continuity and discontinuity of breastfeeding should be studied. Conducted researches around the world show that the factors affecting the breastfeeding include mother's knowledge about the benefits of breastfeeding, mother's attitude to breastfeeding, age at marriage, higher education, family income, received support from the family, decision to breastfeed in pregnancy, experiences during the first lactation, maternal self-confidence, and self-efficacy during the breastfeeding period [10, 11].

In the Bandura's cognitive-social theory, self-efficacy is a cognitive dynamics process that assesses the people beliefs and their ability to conduct a health behavior [12]. One of the predictors of breastfeeding that shows how far the mother is consistent in maintaining breastfeeding and how much she attempts to achieve such a goal is self-efficacy [11]. Self-efficacy on breastfeeding states the following: would the mother choose the breastfeeding? Will the mother try in this regard? And how would she respond to the breastfeeding problems? [13]. When the self-efficacy beliefs are created in people, they will identify their capabilities in different issues and will succeed in changing their self-efficacy beliefs; this will help people effectiveness in addressing the various issues and excite individuals internally to achieve self-actualization by awareness on their intrinsic capabilities [14]. Considering the corrigible nature of self-efficacy, it seems there is chance to create a context for change in maternal perceptions [10, 15].

In a study by Loke and Chan on 199 women in postpartum period using the breastfeeding self-efficacy scale-short form (MBSES-SF) results showed that women with higher score of breastfeeding self-efficacy were more intended to have exclusive breastfeeding six months after birth (OR = 7.77, 95% CI = 2.54, 23.74, *P* < 0.001) [16].

Exclusive breastfeeding is far from WHO recommendation in Iran as well as in Khuzestan Province including Ahvaz. Therefore, the primary aim of this study was to evaluate the effect of educational program on self-efficacy in pregnant women and duration of exclusive breastfeeding after childbearing.

## 2. Subjects and Methods

It was a randomized controlled trial, in which 130 pregnant women over 36 weeks of their first pregnancy recruited. The design of the study was approved by the Ethics Committee of University of Medical Sciences, Tabriz, Iran. The study started in April and was accomplished by December 2010. The inclusion criteria were age over 18 years, having at least basic education, and intention to breastfeed. Women with systemic disease and breast abnormalities were excluded from the study. Among 32 public health care centers in Ahvaz, 11 centers were selected by simple random method. We described the aim of the study for 140 eligible pregnant women and 130 subjects notified their readiness to participate in the study. After obtaining the written consent from each woman, the questionnaires were distributed among them. A questionnaire was prepared to collect sociodemographic data. Breastfeeding self-efficacy scale (BSES) questionnaire consisting of 33 questions was utilized to collect information about breastfeeding self-efficacy before intervention and one month after childbearing [17]. All statements in the Faux and Dennis questionnaire had positive load; each statement had five options from “completely agree” with score 5 to “completely disagree” with score 1. Scores awarded to each statement showed the breastfeeding self-efficacy that the minimum and maximum scores were 33 and 165, respectively. More relevant dimensions to breastfeeding were considered in this questionnaire [17]. The BSES questionnaire was translated to Persian (by two expertises in English) and retranslated to English by a person who was expertise in both English and Persian language. In this research, the content validity method was used to achieve instrument scientific validity. To determine the reliability, a pilot study was performed using 10 eligible subjects. Cronbach's alpha coefficient was calculated as 0.82 representing the internal consistency of the questions. Five questions about breastfeeding continuation were prepared and added to the end of BSES questionnaire. The breastfeeding continuity was assessed six months after childbearing.

### 2.1. Intervention

After completing the initial questionnaires, mothers were randomly divided into two intervention and control groups using randomization.org program. For each individual in the intervention group, a summary of how to hold the meetings, the number of sessions, training materials, and the main objective of the training program was explained. All women in each health center in the intervention group were called and trained in a group. The control group only received routine prenatal care, but participants in the intervention group received two training sessions with two days interval that each session lasted for two hours. Routine prenatal care is containing two items about education of breastfeeding and also a physical examination of breasts that start from the 28th week of gestation. Both groups received this prenatal care.

In the educational sessions, benefits of breastfeeding were explained by a midwife and women were free to ask their questions. Furthermore, a breastfeed mother with previous success in breastfeeding was chosen by the researcher, so that the pregnant mothers could benefit from peer education. Also at these sessions, a training manual feeding guide of breastfeeding was given to pregnant women (the training manual included an introduction about breastfeeding, benefits of breastfeeding for the infant, mother, the community, and the correct positions of nursing, how to properly breastfeed, the positions of the mother in breastfeeding, and methods that increase the mother success in breastfeeding).

During the entire process, all participants in the intervention group were free to contact one of the researchers (SA) in case of experiencing problems in breastfeeding. Persuading and encouraging mothers to breastfeed were performed by one of the researchers (SA) at all steps through phone calls. In addition, every time a mother needed a visit to solve the problem of breastfeeding, the researcher was attended in the health center with previous appointment to solve problems related to the mother's breastfeeding.

### 2.2. Outcome Measures

One month after childbirth, the self-efficacy questionnaires were completed by the study participants. Finally, the questionnaires related to breastfeeding status were completed by all participants six months after child-bearing. Five questions at the end of BSES questionnaire were related to continuation of breastfeeding at the sixth month after delivery, in which the breastfeeding status was categorized in five classes as follow: exclusively breastfeeding, breastfeeding associated with other liquids, breastfeeding associated with less than a bottle (100 mL) of infant formula per day, breastfeeding associated with a bottle of infant formula or more than a bottle per day, and no breastfeeding at all.

### 2.3. Statistics

For data analysis, the statistical SPSS version17 software was used. Descriptive statistics, chi-square, pearson correlation coefficient, paired *t*-test, and independent *t*-test were used for statistical purposes. The relationship or difference is considered significant if *P* was <0.05.

## 3. Results

Ten out of 130 subjects quit the study after evaluating the initial self-efficacy and group dividing. Two subjects in the intervention group before the first training sessions and three subjects after the end of training sessions changed their mind not to participate in the study. The husband of one woman in the control group died and four of them did not provide any reason for withdrawing. The findings showed that the mean age of the samples was 26.6 ± 5.5 years. The average age of marriage was 23.7 ± 2.5 years.

The mean gestational age was 38.6 weeks. There was no significant statistical difference between intervention and control groups in sociodemographic characteristics. The sociodemographic characteristics of participants in two groups are listed in Table 1.

In this study, the mean self-efficacy score in breastfeeding in the last month of pregnancy was 104 in the control group, which did not increase during the first month after delivery. However, the mean self-efficacy score of breastfeeding in the intervention group increased after performing the educational program (Table 2). There was a significant difference between two groups regarding self-efficacy mean difference one month after childbirth (*P* < 0.001).

The results showed a significant difference between the duration of exclusive breastfeeding in intervention and control groups (*P* < 0.001) (Table 2). In addition, there was a significant difference in the continuation of exclusive breastfeeding in the two groups (*P* < 0.001), as 73.3% of the women in the intervention group continued their exclusive breastfeeding up to six months compared to 26.6% in the control group.

Pearson correlation test showed a significant statistical relationship between the self-efficacy score and duration of exclusive breastfeeding (*P* < 0.001). Also there was a significant correlation between the gestational age and self-efficacy of breastfeeding in the women (*r* = 0.12, *P* < 0.05) (results are not shown in the table).

The paired *t*-test showed a significant difference between the mean of self-efficacy scores in the intervention group before and after intervention (*P* < 0.001), while there was no significant difference between the mean of self-efficacy scores in the control group before and after intervention (*P* > 0.05).

## 4. Discussion

This study was aimed to determine the effect of an educational program on self-efficacy in breastfeeding and on exclusive breastfeeding duration in a framework based on Bandura's theory and behavior change model. The study results showed that while self-efficacy changed a little from pregnancy to one month after postpartum in the control group, it increased significantly in the intervention group. Self-efficacy is very important in breastfeeding and is considered as an outstanding variable in determining parameters as choosing breastfeeding, trying to comply and solve the problems encountered during breastfeeding [13].

Self-efficacy is a modifiable and corrigible variable and can be enhanced by implementing an appropriate training program. Breastfeeding self-efficacy is a suitable theoretical framework for directing interventions, which are supposed to be considered for increasing the duration of lactation; in addition, it is a valid instrument for identifying mothers who are at risk of discontinuing of breastfeeding [16].

Hatamleh's study in 2006 on 32 pregnant women showed that the intervention increased the self-efficacy score of breastfeed mothers. In this study, the pretraining self-efficacy score was 108, which increased to 135 after performing the interventional program [18]. This result is consistent with our findings.

The results of our study showed that most of the participants in the intervention group began breastfeeding immediately after delivery and have continued up to the sixth month after delivery. The current study showed that exclusive breastfeeding duration in mothers receiving the educational program increased significantly compared to the control group.

Many studies have been done on exclusive breastfeeding that show the rate of exclusive breastfeeding is still far away from WHO recommendations [5]. Although there is an education regarding exclusive breastfeeding during pregnancy in Iran, due to some reasons it cannot make the great success in breastfeeding. In this study besides midwife's education, the intervention was benefitted from peer education from mothers who had successful experiences in exclusive breastfeeding.

The supportive or unsupportive verbal persuasion of relatives from new mother can increase self-efficacy regarding breastfeeding. However unrealistic percussion can lead to an over inflated perception of self-efficacy and result in mothers' disappointment [17, 19].

A descriptive study conducted by Deborah in 2008 on 70 mothers who had delivered their babies in a hospital located in the Southeast North Carolina. Most of participants have had normal vaginal delivery and a majority of them had breastfeeding experience. Social-cognitive learning theory and self-efficacy had been used in the theoretical framework of this study. Based on this study, there was a significant relationship between self-efficacy scores and exclusive breastfeeding duration, which is in line with the results of our study [20].

Deborah et al. performed a study in Italy to determine the maternal self-efficacy and the perceived insufficiency of breastfeeding. The results suggested that one of the main reasons to discourage mothers from breastfeeding was their low self-confidence [21]. In fact, this study indicates the relationship between self-efficacy and breastfeeding duration, which is in agreement with our study.

A study conducted by Otsuka et al. in Japan showed that there was a significant relationship between breastfeeding self-efficacy and perceived insufficiency of breastfeeding, which is in agreement with our study. Mother or child illness, false beliefs, recommendations of acquaintances and relatives, and reduced breastfeeding self-efficacy are mentioned as factors influencing the nonexclusive breast feeders. Thus, considering all the above factors and developing formulated training programs can be effective in improving the mothers' attitudes and behaviors in relation to exclusive breastfeeding [9].

The results of current study also showed that there was a significant relationship between gestational age and self-efficacy breastfeeding in women so that with increasing the gestational age, the self-efficacy scores of mothers have been reduced. This can be attributed to stress and psychological emotions and the fear of being close to the time of delivery. None of women in this study was teenager.

One of the strengths of this study is that we used peer education to persuade mother to breastfeed. Verbal and social persuasion is the third source of breastfeeding self-efficacy and education and support of relatives and peers are great sources of persuasion and increasing self-efficacy [22]. In a study by Kingston et al. on self-efficacy breastfeeding enhancing using the breastfeeding self-efficacy scale-short form showed that those women who observed breastfeeding role models through videotapes or received supports from their relatives had significantly higher levels of breastfeeding self-efficacy rather than those who received professional assistance [23].

Only small amount of participants in the present study had primary education. We could not educate participants in the intervention group according to their education level; perhaps education according to the participant's literacy level could lead to better results.

## 5. Conclusions

In general, the results showed that the intervention program could increase the self-efficacy of mothers in breastfeeding and the duration of exclusive breastfeeding. Considering the importance of breastfeeding and nutritional benefits of exclusive breastfeeding, the results of this research and statistics available in Iran, especially in Ahvaz, can draw the attention of authorities to the importance of educational programs for mothers regarding the exclusive breastfeeding.

## Figures and Tables

**Table 1:** The Sociodemographic characteristics of intervention and control groups.

Variables	Control group *n* = 60	Intervention group *n* = 60	*P* value
	Mean ± SD or *N* (%)	
Age (y)	26.04 ± 5.4	26.8 ± 5.7	0.91
Age of marriage (y)	23.4 ± 4.9	24 ± 5.4	0.45
Gestational age (wks)	38 ± 2	38 ± 1.4	0.89
Education			
Primary	10 (16.7)	7 (11.7)	0.09
High school degree	19 (31.5)	15 (25)	
Diploma	18 (30.1)	28 (46.7)	
College degree	13 (21.7)	10 (16.6)	
Occupation			
Housekeeper	52 (86.7)	46 (76.7)	0.08
Employed	8 (13.3)	14 (23.3)	
Emotional support			
Very high	10 (16.7)	18 (30)	0.07
High	10 (16.7)	19 (31.7)	
Average	20 (33.3)	11 (18.3)	
Low	17 (28.3)	7 (11.7)	
Very low	3 (5)	5 (8.3)	
Income			
Under 200,0000 T^*♦*^	14 (23.3)	10 (20.3)	0.13
200–500,000 T	24 (40)	23 (39.2)	
>500,000 T	22 (36.7)	27 (40.5)	

^*♦*^Every $ was equal to 1100 Tumans in time of data collection.

**Table 2:** Self-efficacy in breastfeeding before and after childbirth and duration of exclusive breastfeeding in control and intervention groups.

Self-efficacy	Control group *n* = 60	Intervention group *n* = 60	*P* value
	Mean ± SD	
Self-efficacy in the last month of pregnancy	104.4 ± 13.8	105.28 ± 16.3	0.12
Self-efficacy one month after childbirth	101.7 ± 12.19	123.66 ± 12.4	*P* < 0.001
Mean difference of self-efficacy	−2.70 ± 13.7	18.38 ± 12.4	*P* < 0.001
Exclusive breastfeeding (months)	2.73 ± 1.69	5.03 ± 1.6	*P* < 0.001
Exclusive breastfeeding *N* (%)	16 (26.6%)	44 (73.3)	*P* < 0.001
